# Novel inactivation of the causative fungal pathogen of white-nose syndrome with methoxsalen plus ultraviolet A or B radiation

**DOI:** 10.1371/journal.pone.0239001

**Published:** 2020-09-11

**Authors:** Colin J. Hartman, Joseph C. Mester, Patrick M. Hare, Alan I. Cohen

**Affiliations:** 1 Department of Biological Sciences, Northern Kentucky University, Highland Heights, Kentucky, United States of America; 2 Department of Chemistry and Biochemistry, Northern Kentucky University, Highland Heights, Kentucky, United States of America; Vanderbilt University School of Medicine, UNITED STATES

## Abstract

White-nose syndrome is a fungal disease responsible for the rapid decline of North American bat populations. This study addressed a novel method for inactivating *Pseudogymnoascus destructans*, the causative agent of WNS, using ultraviolet A (UVA) or B (UVB) radiation in combination with methoxsalen, a photosensitizer from the furanocoumarin family of compounds. Fungal spore suspensions were diluted in micromolar concentrations of methoxsalen (50–500 μM), then exposed to fixed doses of UVA radiation (500–5000 mJ/cm^2^), followed by plating on germination media. These plates were examined for two to four weeks for evidence of spore germination or inactivation, along with resultant growth or inhibition of *P*. *destructans* colonies. Pretreatment of fungal spores with low doses of methoxsalen resulted in a UVA dose-dependent inactivation of the *P*. *destructans* spores. All doses of methoxsalen paired with 500 mJ/cm^2^ of UVA led to an approximate two-log_10_ (~99%) reduction in spore viability, and when paired with 1000 mJ/cm^2^, a four-log_10_ or greater (>99.99%) reduction in spore viability was observed. Additionally, actively growing *P*. *destructans* colonies treated directly with methoxsalen and either UVA or UVB radiation demonstrated UV dose-dependent inhibition and termination of colony growth. This novel approach of using a photosensitizer in combination with UV radiation to control fungal growth may have broad, practical application in the future.

## Introduction

An alarming expansion of emerging fungal diseases, which pose significant threats to human and animal health, food security, and ecosystem stability, has accompanied widespread anthropogenic activity [1–3]. Management of these diseases is an evolving challenge. Studies of representative mycotic diseases such as White-nose syndrome (WNS) in bats caused by *Pseudogymnoascus destructans* (*Pd)*, and chytridiomycosis in amphibians caused by *Batrachochytrium dendrobatidis* and *Batrachochytrium salamandrivorans*, have documented severe population declines [1, 4]. Recent approaches to limit the spread of *Batrachochytrium salamandrivorans* were able to temporarily contain the fungus but were unsuccessful in eradicating or preventing further dispersal of the disease, emphasizing the need for treatment options [4]. Attempts at treatment have been hindered by potential side-effects on both the host and other organisms in the environment [5, 6]. These emerging pathogens are not limited to the animal kingdom. Rapid Ohia Death, caused by two *Ceratocystis* species, is an emerging fungal disease that is destroying the native Hawaiian tree, Ohi’a lehua (*Metrosideros polymorpha*). There is no effective treatment beyond forest management [7, 8]. New interventions with the capacity to inhibit both fungal hyphal growth and spore germination are urgently needed. This paper presents a novel *in vitro* intervention, using *Pd* as a representative pathogen, for host and environmental treatment with limited non-target consequences.

White-nose syndrome, a recently identified disease caused by *Pd*, has devastated North American bat populations [9–11]. *Pseudogymnoascus destructans* is a psychrophilic filamentous fungus [12–14], with an optimal growth range of 12.5–15.8°C [15]. It requires low to no light [16] and high relative humidity [17]. These conditions are common at sites where susceptible North American bat species winter and enter a reduced metabolic state known as torpor [15]. White-nose syndrome was first recognized in New York State in 2006 and has since decimated many bat populations in North America, with mortality rates of up to 98% (in certain species at specific hibernation sites) [11]. Most US States and Canadian Provinces are either suspected or confirmed to have *Pd* or WNS [9]. White-nose syndrome is characterized by a white fungal growth on muzzles, ears, and wing membranes of bats in torpor [10]. Physiological changes include severe electrolyte disturbance, early arousal from torpor with loss of critical fat reserves, and immune reconstitution inflammatory syndrome, causing significant morbidity and possible host death [9, 18–20]. The damage to affected bat populations is estimated to have resulted in billions of dollars in losses to agricultural communities due to decreased insect control and pollination [9]. Developing effective treatment methods are crucial for preserving the critical role that bats play in North American ecosystems.

While many treatment options have been considered over the past decade, WNS remains an unmitigated threat [5, 21, 22]. Recently published studies have examined management strategies including the use of probiotic bacteria, UVC, polyethylene glycol (PEG), ClO_2_, and vaccines [5, 21, 22]. No vaccine is currently approved for use against any fungal pathogen, including *Pd* [23]. Individual application of common chemical (PEG and ClO_2_) and physical (UVC) disinfectants is considered sub-optimal for controlling infected sites and bats [5]. Applying several of these agents in combination may increase the overall efficacy but may be limited to specific sites or bat species [5]. Chemical disinfectants can be destructive to normal flora, may contaminate local watersheds, and may need repeated application to be effective [5]. Initial studies from our lab indicated that UVC treatment, while effective in inactivating spores of environmental fungi, was limited in its ability to control actively growing fungal colonies, likely due to its poor penetrative ability [24, 25]. No effective treatment option currently exists for controlling *Pd*. There is an urgent need for therapeutics with broad efficacy across sites and species that have limited non-target effects.

Existing chemical or physical control methods may be sub-optimal for controlling *Pd*. A unique combination of psoralens with UVA or UVB radiation may offer an effective treatment option for WNS with limited non-target effects. Photoreactive psoralens are found in nature [26] and can be extracted from plants [26–28] including citrus species, parsnips, parsley, celery, and figs [29]. In human medicine, treatment with the furanocoumarin, methoxsalen (8-methoxypsoralen, 8-MOP), followed by placement of a patient in a dose-controlled UVA light box, is referred to as PUVA (psoralen plus UVA) therapy [30]. PUVA has been used extensively and safely in humans. It is an effective treatment for cutaneous T cell lymphoma, psoriasis, and other skin conditions [26, 31–34]. Ultraviolet A radiation activates methoxsalen, which may generate reactive oxygen species that disrupt cellular components and induce cell death [35–38]. Methoxsalen freely intercalates between cellular DNA strands, causing covalent cross-linking of double-stranded DNA when activated by UVA radiation [39–41]. This cross-linking prevents DNA replication and transcription [42, 43] (S1 Fig). Psoralens alone do not inhibit cellular functions [44], and unlike fungicidal agents such as bleach, should have less impact on the microenvironment of the bat cave [45, 46]. Psoralens degrade quickly under UV light, including sunlight, and are expected to degrade rapidly by reactions with atmospheric hydroxyl radicals and under alkaline conditions, but otherwise are modeled to persist in soil for weeks [47, 48]. This persistence is not predicted to be of concern because of the low toxicity of methoxsalen [49] and may allow for flexibility in cave treatment methods. Ultraviolet A and UVB radiation have lower energy profiles compared to UVC radiation, which may be advantageous for treating bats and their microenvironment [35]. Combining lower energy UV wavelengths with psoralens would allow for the ability to treat bats in a similar manner to PUVA therapy to reduce the *Pd* burden and allow for a more appropriate immune response. Treating the cave environment with this novel approach may limit impact on other flora and fauna, especially when compared to other disinfectants [45].

The primary objective of this study was to determine whether methoxsalen plus UVA at 365 nm or UVB at 312 nm had fungicidal or fungistatic effects against *Pd*. Spores from *Pd* were chosen because they populate and persist in the cave environment and serve as the infectious agent of WNS [3]. Fungal spores are considered to have a survival advantage versus vegetative colonies in adverse environmental conditions. In addition, *Pd* hyphal growth was examined to determine the effect of methoxsalen plus UVA or UVB exposure on actively growing colonies. Methoxsalen-infused media and topical application of methoxsalen were used to assess the efficacy of the treatment on germinating spores and established colonies. The concentrations of methoxsalen and the doses of UV utilized in this study were chosen based on prior *in vivo* studies in humans [50, 51] and our preliminary unpublished *in vitro* data. The results of this study demonstrated that methoxsalen plus UVA or UVB treatment had fungicidal and fungistatic effects against *Pd* that may be applicable to bats and their environment.

## Materials and methods

### *P*. *destructans* spore suspensions, methoxsalen, and UV exposure

*Pseudogymnoascus destructans*, isolated from the wing skin of the little brown bat (*Myotis lucifugus*), was obtained from the American Type Culture Collection (ATCC® MYA4855™) and stored at -80°C. Portions of the vial, containing viable hyphae and spores, were thawed and plated on potato dextrose agar (PDA, Difco). For *Pd* culture, all inoculated plates were covered with aluminum foil to protect against light and incubated at 10–15°C for 2 weeks. Spore suspensions were prepared in sterile phosphate-buffered saline solution containing 0.05% Tween20 (PBS-Tw20, MilliporeSigma) by adding approximately 10 ml of solution per plate and collecting the spores with an L-shaped spreader. Spore suspensions were filtered through sterile cheesecloth to minimize hyphal contamination. Spore enumeration was performed using a hemocytometer (Bright-Line™). Suspensions were diluted with PBS-Tw20 to approximately 5 x 10^6^ spores/ml, stored at 4°C, and protected from ambient light.

Liquid methoxsalen was harvested from 10 mg VRX 650 capsules (Valeant Pharmaceuticals North America), diluted initially in glacial acetic acid, and then in PBS-Tw20, to final methoxsalen concentrations of 50–500 μM. Final acetic acid concentrations for spore and colony treatments ranged from 4–40 mM. For spore treatments with methoxsalen, spore suspensions of 1x10^6^/ml were prepared in approximately 0.5 ml final volumes. Spore solutions were exposed to UV in volumes of 100 μl. For spectroscopy, purified methoxsalen (99% 8-methoxypsoralen, Alfa Aesar) was dissolved in glacial acetic acid and diluted to 500 μM in PBS-Tw20. The UV-Vis absorption spectrum of the VRX 650 methoxsalen in PBS-Tw20 and 40 mM acetic acid was measured in a Shimadzu UV-2600 spectrophotometer following subtraction of a PBS-Tw20 and acetic acid blank in 1 cm pathlength fused silica cuvettes. The spectrum of purified methoxsalen in PBS-Tw20 and acetic acid was also measured for comparison.

Measured doses of UV radiation (in mJ/cm^2^) were administered using a Stratagene Stratalinker® 2400 UV Crosslinker (Agilent Technologies) containing an array of 5 mercury vapor bulbs for UVA (365 nm) or UVB (312 nm).

### *P*. *destructans* spore treatment with methoxsalen followed by exposure to UVA

*Pseudogymnoascus destructans* spore treatments were conducted as biological quadruplicates using methoxsalen concentrations of 50, 250, and 500 μM, and UVA doses of 500, 750, and 1000 mJ/cm^2^. Spore suspensions were exposed to specific methoxsalen concentrations for 20–24 hours at 15°C or for 15, 30, and 60 minutes at room temperature prior to UVA exposure. Control spore solutions were exposed to either UVA only, methoxsalen only, or left untreated. All spore control and treatment suspensions were established at a final concentration of approximately 1 x 10^6^ spores/ml. Aliquots of 100 μl of treatment and control suspensions were spot plated in the center of 60 x 15 mm Petri dishes and exposed to their assigned doses of UVA. Afterwards, utilizing an 8 x 12 deep well microtiter plate, a four-step, 1:10 dilution series was conducted by adding 40 μl of each spore suspension to 360 μl of PBS-Tw20 to produce a resultant 400 μl solution. Forty microliter aliquots from these dilutions were spread over half of a PDA plate with a sterile loop. The limit of detection for this assay was 250 colony forming units (CFU)/ml. The plates were incubated at 15°C using aluminum foil to eliminate ambient light. Colony growth was observed at days 10 through 18 post-plating. Colony counts were performed when colonies reached approximately 5 mm in diameter. Dilution plates with colony counts ranging from 30–300 per plate were used to calculate total spore viability in CFU/ml for control and treatment groups.

### *P*. *destructans* spore germination on methoxsalen-infused media followed by UVA exposure

Methoxsalen-infused media was prepared by adding 500 μM methoxsalen in PBS-Tw20 to PDA plates for a final concentration of 15 μM per plate. Needle inoculation of *Pd* spores was performed to obtain four actively growing colonies per plate. When the colonies were approximately 5 mm in diameter (approximately 9–10 days later) a single dose of UVA was administered. Control colonies on methoxsalen-free media received either UVA or no UVA, and additional control colonies on methoxsalen-infused media received no UVA. Resultant radial colony growth was measured every three days with a scientific ruler. All plates were incubated at 15°C in the absence of light.

### *P*. *destructans* colony infusion with methoxsalen and UVA or UVB exposure

Needle inoculation of *Pd* spores was performed to obtain four actively growing colonies on each PDA plate. When the colonies were approximately 5 mm in diameter (approximately 9–10 days later), they were infused with a 500 μM methoxsalen solution by directly pipetting the methoxsalen onto the surface of the colony (1 μl/mm^2^ of colony size). Single dose experiments were performed with one methoxsalen treatment followed immediately by a single UVA exposure. Multiple dose experiments were performed by re-treating colonies with methoxsalen and fixed doses of UVA or UVB every four days. Each experimental treatment group was composed of eight colonies growing on two plates (four colonies per plate).

### Statistical analyses

All statistical analyses were performed using non-transformed colony count data using SPSS® Statistics version 26. Analysis of variance (ANOVA) was utilized for data from more than two treatment groups. One-way ANOVA with Bonferroni post hoc tests were utilized to determine significance between means of experimental groups. Two-way ANOVA was used for data with two treatment variables, and simple main effects tests were utilized to determine significance between individual groups. Repeated measures ANOVA (RMA) with Bonferroni post hoc tests were utilized to compare means across various time points. All statistical procedures utilized a 95% confidence interval (α = 0.05).

## Results

### Treatment of *P*. *destructans* spores with methoxsalen and UVA

**Fig 1:**
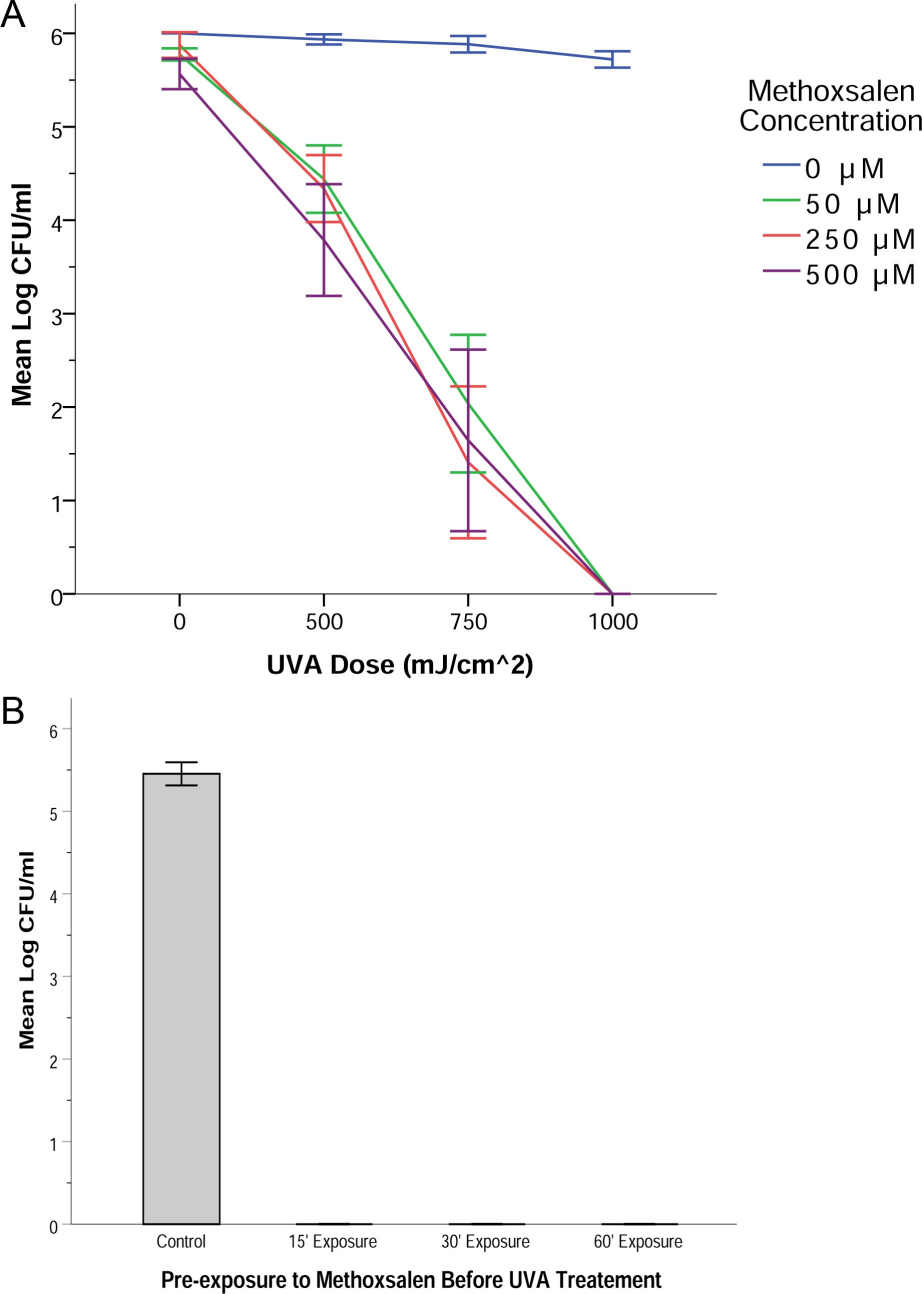
*Pseudogymnoascus destructans* sporicidal activity of UVA in combination with methoxsalen. (A) Increased doses of UVA had increased sporicidal effect against *P*. *destructans* when paired with methoxsalen pre-treatment for 20–24 hours. The sporicidal effect of UVA appeared to be log-linear when paired with 50–500 μM methoxsalen. (B) Methoxsalen pre-treatment (500 μM) for 15, 30, or 60 minutes followed by UVA exposure (1000 mJ/cm^2^) yielded no detectable spore germination. Results shown in (A) and (B) were derived from four replicates. The limit of detection was 250 CFU/ml. Error bars represent one standard error of the mean.

Treatment with UVA or methoxsalen alone did not yield a *Pd* sporicidal effect (Fig 1A and S2 Fig). Spore solutions that received UVA exposure after a 20–24 hour methoxsalen exposure, however, showed significant sporicidal effects. There was a significant interaction between the effects of the dosage of UVA and the concentration of methoxsalen (two-way ANOVA, *F*_9,48_ = 8.606, *p* < 0.001) (Fig 1A and S2 Fig). Simple main effects tests showed that there was a significant sporicidal effect between the treatment groups and all control groups (all p < 0.05). All methoxsalen concentrations tested (50, 250, and 500 μM) had an equivalent sporicidal effect when combined with different doses of UVA (Fig 1A and S2 Fig). As the dose of UVA increased, the number of surviving spores (mean log_10_ CFU/ml after plating) decreased (Fig 1A and S2 Fig). All doses of methoxsalen paired with 500 mJ/cm^2^ of UVA led to an approximate two-log_10_ (~99%) reduction in spore viability, and when paired with 1000 mJ/cm^2^, a four-log_10_ or greater (>99.99%) reduction in spore viability was observed (Fig 1A). Treatment groups receiving 750 mJ/cm^2^ of UVA with methoxsalen had a few detectable colonies after plating, near the limits of detection in our assay (250 CFU/ml). Treatment groups receiving 1000 mJ/cm^2^ of UVA with methoxsalen yielded no detectable colonies (Fig 1A and S2 Fig). Exposure of *Pd* spores to methoxsalen (500 μM) for 15, 30, and 60 minutes followed by UVA exposure (1000 mJ/cm^2^) produced a significant sporicidal effect (Fig 1B). Treatment groups receiving both methoxsalen and UVA exposure yielded no detectable colonies. There was a significant difference between experimental groups (one-way ANOVA, *F*_3,30_ = 1190.3, *p* < 0.001), and Bonferroni post hoc comparisons indicated that spores treated with methoxsalen for 15, 30, and 60 minutes prior to UVA exposure exhibited significantly less spore germination than control spores (all *p* < 0.001).

### UVA exposure on methoxsalen-infused media

**Fig 2:**
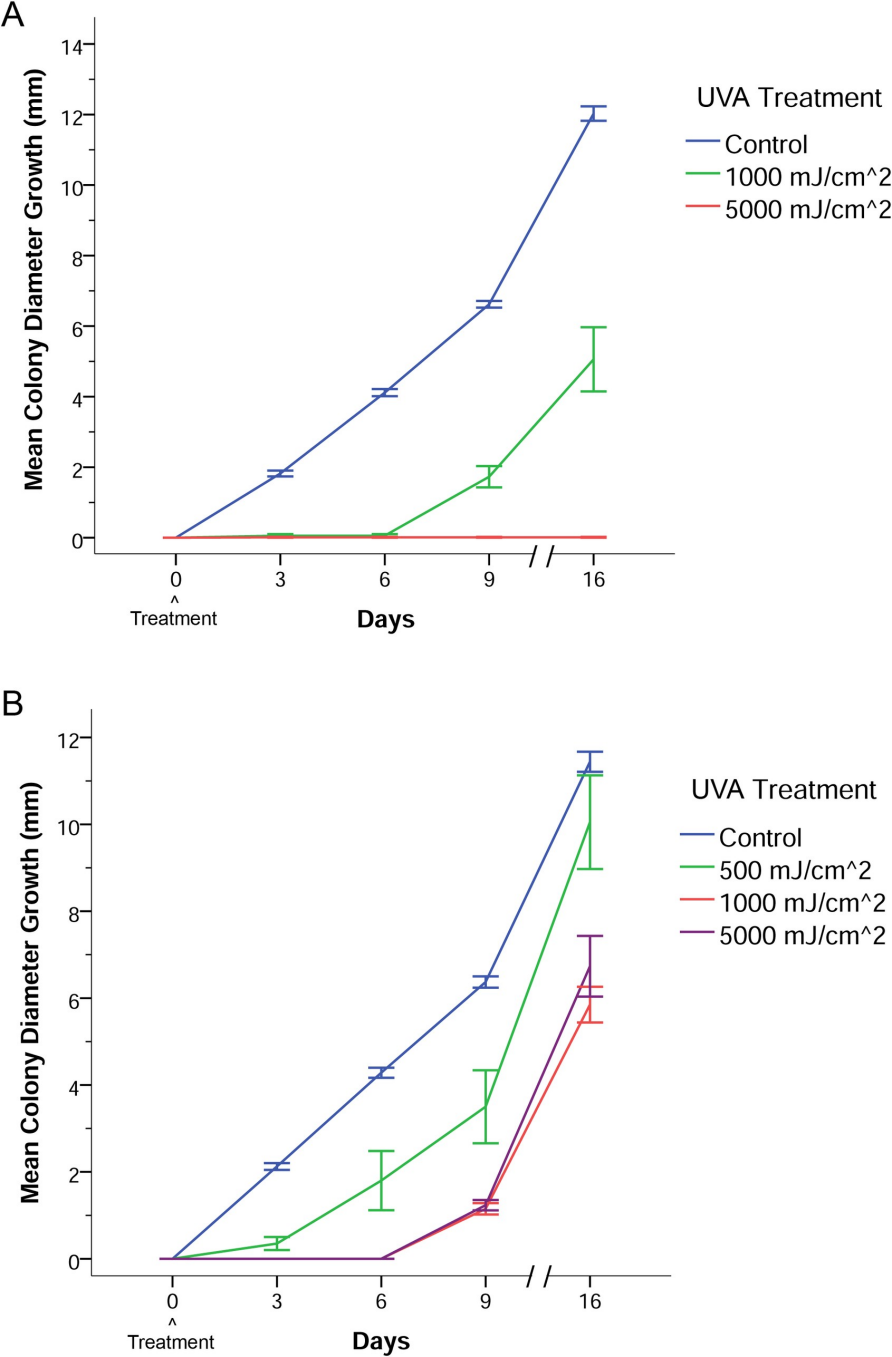
*Pseudogymnoascus destructans* colony growth after methoxsalen treatment and a single UVA exposure. (A) Ultraviolet A exposure inhibited colony growth of *P*. *destructans* in a dose-dependent manner on methoxsalen-infused media (15 μM final). Control colonies received either UVA (1000 or 5000 mJ/cm^2^) on methoxsalen-free media, no UVA on methoxsalen-infused media, or no UVA on methoxsalen-free media. Results were derived from six to eleven biological replicates per data point. Error bars represent one standard error of the mean. (B) A single UVA dose inhibited growth of *P*. *destructans* colonies topically infused with a methoxsalen solution (1 μl of 500 μM methoxsalen per mm^2^ of colony size) immediately before UVA exposure. Results were derived from four biological replicates. Control colonies received no UVA or methoxsalen or were singly exposed to either UVA or methoxsalen. Error bars represent one standard error of the mean.

To determine if methoxsalen plus UVA treatment would impact actively growing colonies of *Pd*, spores of *Pd* were germinated on methoxsalen-infused media and given single doses of UVA radiation. Colonies of *Pd* grown on methoxsalen-infused media (15 μM final) and treated with UVA demonstrated a significant inhibition of radial colony growth compared to control colonies (RMA, *F*_2,43_ = 604.36, *p* < 0.001) (Fig 2A). Control colonies received either UVA exposure (1000 and 5000 mJ/cm^2^) on methoxsalen-free media or no UVA exposure on methoxsalen-infused media. Bonferroni post hoc comparisons indicated that the mean radial growth of *Pd* colonies grown on methoxsalen-infused media and irradiated with UVA at 1000 mJ/cm^2^ (*p* < 0.001) or 5000 mJ/cm^2^ (*p* < 0.001) was significantly different from control colony growth. Control colonies grown on methoxsalen-infused media only, or colonies exposed to UVA only, did not have a significant difference in mean radial colony growth compared to colonies grown on methoxsalen-free media that received no UVA exposure (*F*_3,25_ = 1.24, *p* = 0.316). Control colonies demonstrated an approximate growth rate of 0.75 mm/day, while those grown on methoxsalen-infused media that received a single dose of UVA at 1000 mJ/cm^2^ demonstrated an approximate growth rate of 0.32 mm/day (Fig 2A). Colonies of *Pd* on methoxsalen-infused media that received a 5000 mJ/cm^2^ dose of UVA demonstrated no growth (Fig 2A).

### Direct colony treatment with methoxsalen followed by UVA

Topical application of methoxsalen on actively growing colonies (1 μl of 500 μM methoxsalen per mm^2^ of colony size) followed by a single UVA dose inhibited *Pd* growth. Colonies exposed to methoxsalen and 500 mJ/cm^2^ UVA demonstrated slower radial growth between days 1 and 9 post-treatment than control colonies (Fig 2B). With UVA doses of 1000 and 5000 mJ/cm^2^, colonies did not exhibit radial growth until day 7, and growth on days 7 through 9 was slower than that of untreated controls (Fig 2B). There was a significant difference due to UVA exposure on radial *Pd* colony growth between the experimental groups (RMA, *F*_3,32_ = 117.23, *p* < 0.001). Bonferroni post hoc comparisons indicated that the mean radial growth of *Pd* colonies exposed to methoxsalen and UVA at 500 mJ/cm^2^, 1000 mJ/cm^2^, and 5000 mJ/cm^2^ were all significantly different (*p* < 0.001) from the control mean radial growth. An RMA was also performed on the methoxsalen and UVA only control data. The colonies treated with either methoxsalen or UVA did not have a significant difference in mean radial colony growth versus colonies not exposed to either methoxsalen or UVA (*F*_4,19_ = 1.54, *p* = 0.232).

### Repeated methoxsalen and UVA treatment of *P*. *destructans* colonies

**Fig 3:**
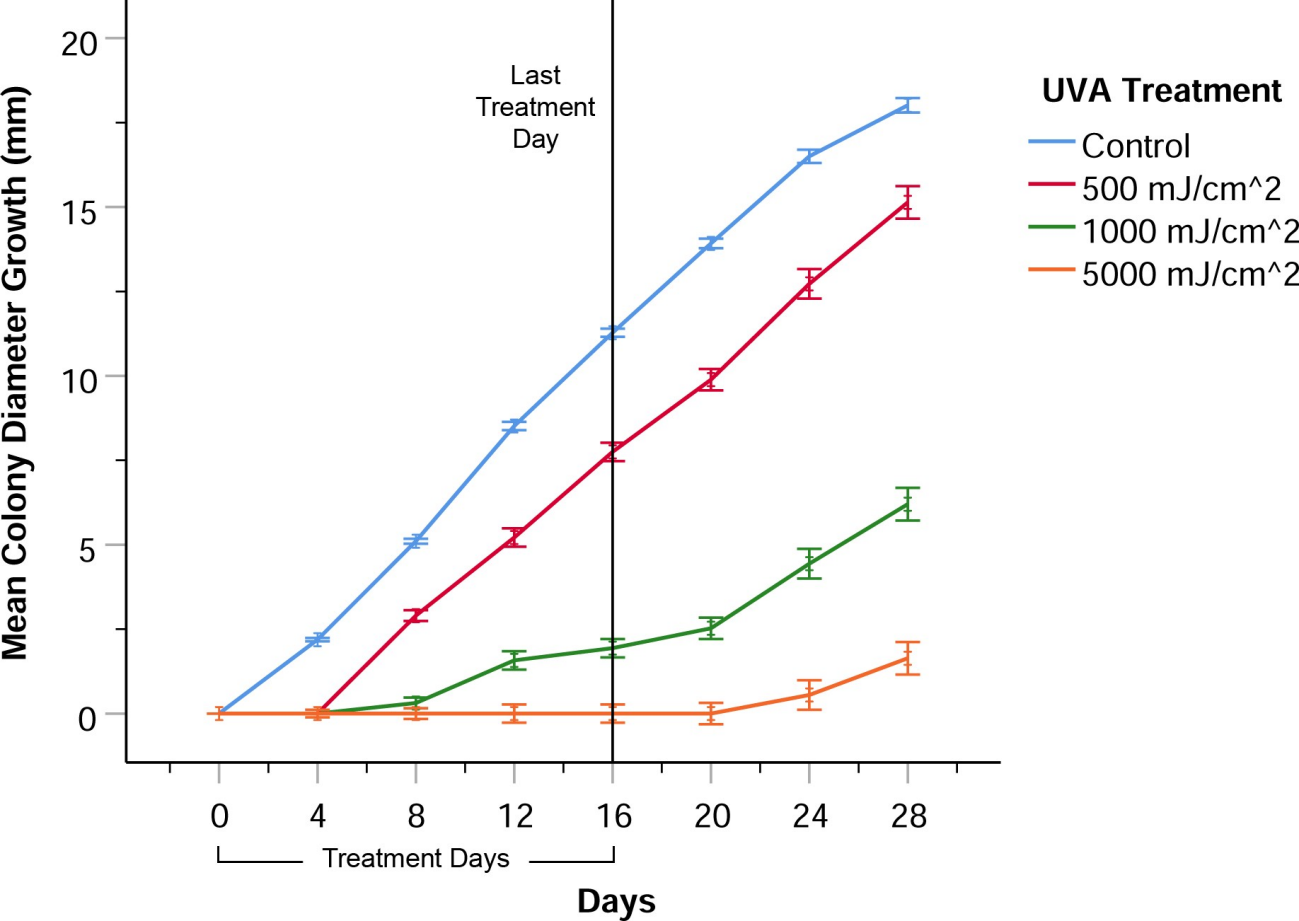
*Pseudogymnoascus destructans* colony growth with repeated methoxsalen and UVA exposure. Colonies were exposed to methoxsalen and UVA (365 nm) once every four days, for a total of five treatments. Ultraviolet A exposure inhibited growth of *P*. *destructans* colonies infused with methoxsalen in a UV dose-dependent manner. Control colonies received no UVA exposure or methoxsalen, or were exposed to either UVA or methoxsalen. Results were derived from eight biological replicates. Error bars represent one standard error of the mean.

To further explore the anti-fungal effects of methoxsalen plus UVA, a regimen of topical methoxsalen instillation onto actively growing colonies of *Pd* followed immediately by UVA exposure was repeated every four days. With this regimen, methoxsalen-exposed colonies receiving a 500 mJ/cm^2^ dose of UVA radiation demonstrated an initial inhibition of radial growth after the first treatment, but further growth was not inhibited (Fig 3). Methoxsalen-exposed colonies receiving a 1000 mJ/cm^2^ dose of UVA radiation demonstrated continual inhibition of growth with each treatment versus control colonies, but the amount of this inhibition decreased 4 days after the treatments ended (Fig 3). Methoxsalen-exposed colonies receiving 5000 mJ/cm^2^ of UVA radiation demonstrated the highest level of growth inhibition. Colonies only began to grow radially, at a slow rate, 5 to 8 days after the last treatment (Fig 3). Images of *Pd* colonies at day 16 after repeated methoxsalen and UVA treatment are shown in the S3 Fig.

There was a significant effect of UVA exposure on radial *Pd* colony growth in the experimental groups (RMA, *F*_3,60_ = 770.02, *p* < 0.001). Bonferroni post hoc comparisons indicated that the mean radial growth for colonies infused with methoxsalen and irradiated with UVA at 500 mJ/cm^2^, 1000 mJ/cm^2^, and 5000 mJ/cm^2^ were all significantly different (*p* < 0.001) from the control mean radial growth. Bonferroni post hoc comparisons indicated that the mean radial growth was not significantly different between untreated, UVA only, or methoxsalen only control groups (*p* > .05).

### Repeated methoxsalen and UVB treatment of *P*. *destructans* colonies

The UV absorption spectra of methoxsalen extracted from the VRX 650 capsules and purified methoxsalen (99% 8-methoxypsoralen) were initially determined to assess their similarity. There was no significant difference in UV absorption between the two sources of methoxsalen at the wavelengths used in this study (S4 Fig). Both solutions demonstrated broad absorption in the UV range, with peaks at 305 nm and 247 nm, in agreement with previous literature [16, 36, 52]. The spectra also suggested that UVB wavelengths were suitable for exciting methoxsalen, and that they may have a combined inhibitory effect on *Pd*.

**Fig 4:**
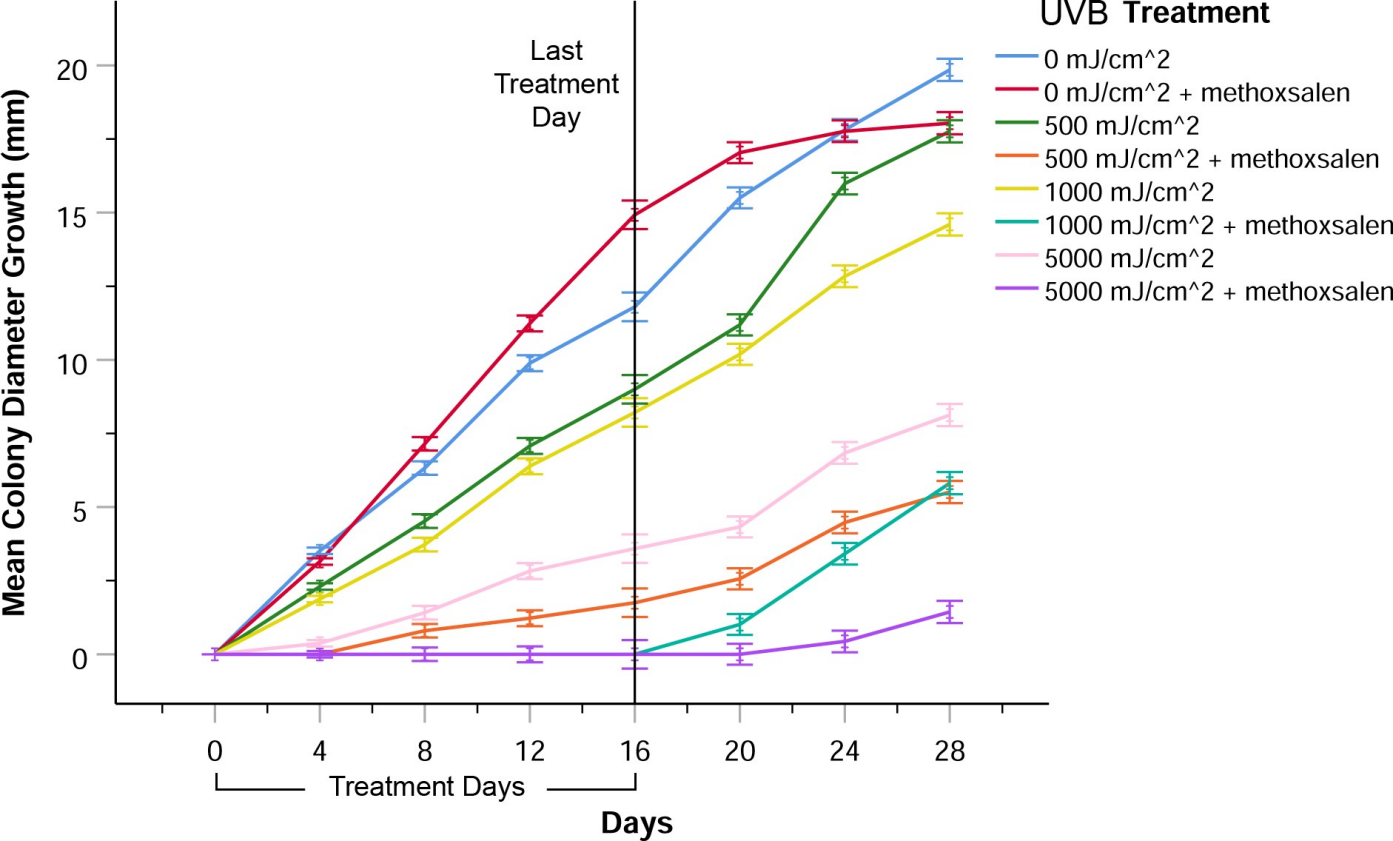
*Pseudogymnoascus destructans* colony growth with repeated methoxsalen and UVB exposure. Colonies were exposed to methoxsalen and irradiated with UVB (312 nm) once every four days, for a total of five treatments. Control colonies received no UVB exposure or methoxsalen, or were singly exposed to methoxsalen. Colonies receiving UVB alone demonstrated growth inhibition. Ultraviolet B exposure inhibited growth of *P*. *destructans* colonies infused with methoxsalen in a UV dose-dependent manner. Results were derived from eight biological replicates. Error bars represent one standard error of the mean.

To examine the anti-fungal effects of methoxsalen combined with UVB (312 nm), a regimen of methoxsalen instillation onto actively growing colonies of *Pd* followed immediately by UVB exposure was repeated every four days. Colonies receiving 500 mJ/cm^2^ of UVB radiation alone demonstrated a slight inhibitory effect on colony growth (Fig 4). Higher doses of UVB, 1000 mJ/cm^2^ and 5000 mJ/cm^2^, also inhibited colony growth without methoxsalen pre-treatment. Exposure to methoxsalen before UVB treatment greatly enhanced the inhibitory effect of each UVB dose on colony growth (Fig 4). Combining methoxsalen with the lowest dose of UVB (500 mJ/cm^2^) resulted in greater colony inhibition than the highest dose (5000 mJ/cm^2^) of UVB alone. Methoxsalen-exposed colonies receiving either 1000 mJ/cm^2^ or 5000 mJ/cm^2^ of UVB radiation demonstrated the greatest inhibition of growth, where colonies only began to grow radially after the last treatment (Fig 4). Images of *Pd* colonies at day 24, after repeated methoxsalen and UVB treatment, are shown in the S5 Fig.

There was a significant difference due to UVB exposure on radial *Pd* colony growth among the experimental groups (RMA, *F*_6,57_ = 486.53, *p* < 0.001). Bonferroni post hoc comparisons indicated that the mean radial growth for colonies infused with methoxsalen and irradiated with UVB at 500 mJ/cm^2^, 1000 mJ/cm^2^, and 5000 mJ/cm^2^ were all significantly different (*p* < 0.001) from the control mean radial growth. Individual comparisons of treatment groups that received only UVB and their counterparts that received methoxsalen exposure with the same dose of UVB indicated that there was a significant difference in mean radial growth between each pair of treatment groups (500 mJ/cm^2^, *p* < 0.001; 1000 mJ/cm^2^, *p* < 0.001; 5000 mJ/cm^2^, *p* < 0.001). There was a significant difference in mean radial colony growth (RMA, *F*_4,35_ = 4.79, *p* < 0.001) between the control groups that received no UVB exposure or methoxsalen, or were singly exposed to either UVB or methoxsalen. Bonferroni post hoc comparisons confirmed that the mean radial growth was significantly different between the control groups receiving no UVB and those receiving UVB but no methoxsalen (*p* < 0.001).

## Discussion

*Pseudogymnoascus destructans*, a fungal pathogen first recognized in Northern American bat caves in 2006, has caused a significant depletion of select bat populations [11]. Experimental treatments and vaccines are being studied to limit the spread and mortality of bats due to WNS, yet none have found widespread application [5]. This study demonstrated a novel treatment method for controlling *Pd* growth. Exposure to methoxsalen, followed by UVA or UVB exposure, inactivated *Pd* spores and inhibited vegetative growth *in vitro*. While neither UVA nor methoxsalen treatment alone had a significant effect on the viability of fungal spores or colonies, the combination of UVA plus methoxsalen demonstrated inhibitory and fungicidal effects on *Pd*. Dormant spores, germinating spores, and actively growing colonies of *Pd* were all significantly impacted by this regimen. The extent of the inhibitory/fungicidal effect was UV dose-dependent. A single dose of UVA and methoxsalen led to a four-log_10_ reduction in spore viability. Single-dose combined treatment of germinating spores eliminated colony growth. Repeated administration of methoxsalen and UVA was able to completely inhibit *Pd* colony growth. Thus, treatment of *Pd* with UVA after methoxsalen treatment elicited robust *in vitro* fungistatic and fungicidal effects. This approach could be adapted to the treatment of *Pd* infected bats and their cave environment.

Methoxsalen appeared to be rapidly taken up by both fungal spores and actively growing hyphae. A brief 10–15 minute pre-exposure to methoxsalen was sufficient to yield notable sporicidal and fungicidal effects with UVA. Higher doses of UVA, and repeated UVA exposures, were needed for fungicidal activity against growing colonies, whereas single low doses of UVA were effective against spores. Fungal spores are metabolically dormant, while living cells possess DNA repair enzymes capable of correcting damage to cellular DNA. A previous report noted that *Pd* is lacking a component of the alternative excision repair pathway but did acknowledge that the *Pd* genome has evidence of enzymes involved in the base excision repair and nucleotide excision repair pathways [16]. Active repair of psoralen DNA photoadducts and oxidative DNA lesions likely contributes to protection and explains the higher doses of UVA needed to control fungal colonies versus spores.

There is limited information in the literature regarding the efficacy of combining psoralens with UVB [52]. Methoxsalen’s absorption is significantly stronger in the UVB than the UVA spectrum, potentially leading to more efficient photoactivation [36]. Our results demonstrated that UVB was able to activate methoxsalen and inhibit the growth of *Pd*. Ultraviolet B treatment alone had a dose-dependent inhibitory effect on *Pd* growth. The combination of methoxsalen with any of the three doses of UVB that were examined led to significantly more inhibition than the highest dose of UVB (5000 mJ/cm^2^) alone. Repeated administration of methoxsalen with either 1,000 or 5,000 mJ/cm^2^ UVB was able to completely inhibit *Pd* colony growth. With methoxsalen pre-treatment, the amount of colony growth inhibition seen following the different UVB doses was approximately the same as that seen with similar UVA doses.

Methoxsalen plus UVA, with its longer wavelength, may be a preferred approach over methoxsalen combined with UVB or UVC. Ultraviolet B and UVC wavelengths are fungicidal [53, 54] and UVC, *in vitro*, is fungicidal for *Pd* [16]; however, these wavelengths have limitations due to their higher energy and carcinogenic effects versus lower energy UVA [18]. The longer wavelengths of UVA, compared to UVB and UVC, permit deeper penetration to the dermis where *Pd* invades [20, 55].

Control of the spread of WNS, with selective treatment of infected bats and contaminated caves, will rely on methods that are both effective at inhibiting *Pd* growth and limiting non-target effects. Individual applications of disinfecting chemicals or UVC light are frequently used for surface decontamination [46, 56–58]. These chemicals and UVC light, however, can be corrosive or damaging to exposed surfaces, harmful to many organisms in the environment, and caustic when contacting mucous membranes or skin [46, 59, 60]. As demonstrated here, combining two minimally toxic modalities—non-ionizing, long-wavelength UVA or UVB light with methoxsalen—produced a potent fungicidal effect. Each agent potentiated the effect of the other, allowing minimal doses of each agent to achieve the maximum possible effect. This should allow for overall lower concentrations of psoralens to be utilized than other stand-alone agents. With this method, methoxsalen was effective at concentrations of 0.001–0.01%, whereas bleach (NaOCl) is typically utilized at a 0.05–0.5% final concentration [61]. The limited toxicity of a single agent by itself allows for a broad application of one treatment component, and a more targeted application of the second component. This offers better control and targeting while minimizing non-target effects. Psoralens plus UVA or UVB, unlike currently available fungicidal agents, could have less impact on the microenvironment of the bat cave [45–49]. Anticipated non-target effects in bats based on clinical experience in humans include phototoxic erythema and brief nausea [62, 63]. Cumulative UVA exposures would raise concerns of melanoma and squamous cell carcinomas in these mammals [32]. Methoxsalen alone is not mutagenic [49] but may be mutagenic in combination with UVA exposure [48, 49, 64]. There is no evidence to suggest that PUVA therapy is a potent teratogen [64]. Other non-target effects may include detriment to microbiota at bat hibernacula within the targeted areas [36, 65]. Potential application of methoxsalen and UVA or UVB in the environment will require a holistic approach to the ecosystem.

Innovative approaches can be developed to adapt delivery methods for the field or cave environments. The ability to deliver this photosensitizer as a food source for bats is being studied by our group. Psoralens are found in dates, figs, and many other naturally occurring substances [29]. This may provide a non-invasive method to establish therapeutic levels of psoralens in bats requiring directed treatment. Methoxsalen levels peak in tissues 0.5–4 hours after ingestion, and metabolites appear in the urine after 8 hours [63]. Multiple brief UV treatments of bats severely infected by *Pd* would limit local adverse skin effects from UV.

Portable, low energy, high efficiency sources of UVA or UVB will be required for this system to be deployed in remote bat hibernacula. Recent advances in light-emitting diode (LED) technology can be adapted for this use [66]. This approach would permit tailoring of the UV wavelength to that most effective for psoralen activation, as action spectra often do not track absorption spectra exactly [67]. The pulsing of UVA or UVB LEDs may result in a lower total power (energy/time) required for spore and colony inactivation. Preliminary studies performed in our lab support the potential for this technology to replace the use of large, inefficient mercury vapor bulbs for UV delivery (unpublished data).

Bat tissues infected with *Pd* and cave walls colonized with *Pd* fluoresce in the presence of UVA light, allowing for easy detection and focused treatment [68]. Topical methoxsalen application to *Pd* contaminated bats (identified by UVA fluorescence) prior to their coming out of torpor may limit the destructive immune response to the fungus (immune reconstitution inflammatory syndrome). A field UV emitter could be powered by a small, 12-volt, lithium iron phosphate battery attached to a microcontroller with associated photoresistors to turn the UV emitters on and off. Methoxsalen sensitized bats entering or leaving their roosts would activate the devices producing an instant pulse of UV light. These could be calibrated based on future studies determining the dose of UVA or UVB required to suppress *Pd* colonization. Similarly, bat roosts could be sprayed or misted with a methoxsalen containing reagent, followed by irradiation with UVA or UVB via a pulse width modulating microcontroller attached to a specific wavelength UV emitter. Adding a photoresistor would allow for *Pd* colonizes to be treated during times that bats are outside of their roosts. Unlike UVC, the longer wavelengths associated with UVA and UVB should result in deeper photon penetration into the dermis of affected bats and potentially deeper in cave soil and guano. The expected persistence time of weeks for psoralens in soil [47] could provide flexibility in the timing between methoxsalen treatment and the delivery of UV light, as well as permitting a longer time frame for treatment. Applying well established PUVA time-based dose sequencing techniques that have been utilized in human medicine should limit non-target bat toxicity such as erythroderma and avoid approaching carcinogenic doses [69].

A concurrent study performed in our lab using *Penicillium crustosum* and other environmental fungi supports the broader application of this novel intervention. That study demonstrated excellent fungicidal effect using methoxsalen plus UVA [70]. Based on these initial pilot studies, this unique combination of UV plus methoxsalen may have broader applications for controlling other emerging fungal infections such as chytridiomycosis and Rapid Ohia Death [1, 7]. Methoxsalen was effective at low concentrations and was taken up quickly by living cells and spores. Ultraviolet A is penetrative to soil and tissue and minimally damaging on its own. This method allows for precise dosing in the amount and frequency of psoralen application, and the amount and frequency of UVA exposure. This approach has advantages over harmful disinfectants such as bleach which is deleterious to amphibians when used to control chytridiomycosis [45]. Amphibians with chytridiomycosis could be treated by applying methoxsalen topically or through oral ingestion, followed by selective UV dosing schedules. Methoxsalen applied daily for 21 days to the plant *Citrus sinensis*, in the presence of ambient sunlight, did not have a detrimental effect to the leaf tissue [36]. Trees afflicted by fungal diseases such as Rapid Ohia Death could be treated with a methoxsalen topical spray or spike delivery system followed by UV exposure. Studies will be required to determine the best vehicle and appropriate dose of methoxsalen. Depth of penetration of UV light into leaf and vascular tissues will need to be examined on a species-specific basis. Outdoor application would also have to account for ambient UVA and UVB levels from sunlight, which would likely minimize the required therapeutic amount of device-delivered UV [36].

Treatment of *Pd* with UVA or UVB in combination with methoxsalen resulted in robust *in vitro* fungistatic and fungicidal effects. Unlike UVC, UVA and UVB are non-ionizing, and due to their longer wavelength penetrate deeper into the dermis [55], where *Pd* is found. Additional preliminary studies from our lab demonstrated similar effects against other fungal species. This method has potential *in vivo* application both for the treatment and containment of the fungus causing WNS and for other emerging environmental fungal pathogens. Recent advances in LEDs have created energy efficient, portable, and directional options for specific UV emissions. Light-emitting diode devices with specific wavelengths can be prototyped for targeted treatment of infected bats or for broader environmental containment applications. The concentration of methoxsalen required to produce therapeutic effect in combination with UVA or UVB is significantly less than other environmental fungicidal agents such as bleach. Based on years of experience in treating humans with both systemic and topical methoxsalen, toxicity associated with this approach was limited and non-target effects were easily minimized through careful dosing. This method will need to be studied *in vivo* in bats. This novel combination of UVA or UVB plus methoxsalen may be applicable to other rapidly emerging fungal diseases, for which current interventions are limited or unavailable.

## Supporting information

S1 Fig Cross-linking activity of methoxsalen. (A) Intercalation of methoxsalen between DNA base pairs. (B) Covalent crosslinks form when activated by UV radiation.
(TIF)

S2 Fig Decreased viability of *Pseudogymnoascus destructans* spores treated with methoxsalen and UVA. Spore germination, resulting in *P*. *destructans* colony formation, was observed after various treatments. Each set of 2 rows represents spore dilutions of 10^−2^ to 10^−5^ split across two plates. Each column shows spores exposed to a specific UVA dose. Each set of 2 rows represents spores pre-treated with a specific amount of methoxsalen (the first 2 rows show spore controls that were not pre-treated with methoxsalen). Spores were suspended in various concentrations of methoxsalen for 20–24 hours before UVA exposure. Spore inactivation was evident for all spores that were treated with both methoxsalen and UVA. This image was taken 11 days after spore plating.
(TIF)

S3 Fig Colony growth of *Pseudogymnoascus destructans* after repeated methoxsalen and UVA treatment. These plates correspond to the experimental results shown in Fig 3. Each image was taken 16 days after the initiation of treatment. The black circles at the center of each colony represent the original colony diameter immediately before the first treatment. The control plate shows normal colony growth.
(TIF)

S4 Fig Methoxsalen absorbance comparison. Solvent-subtracted normalized absorption spectra of methoxsalen extracted from VRX 650 capsules (red) and purified methoxsalen powder (blue).
(TIF)

S5 Fig Colony growth of *Pseudogymnoascus destructans* after repeated methoxsalen and UVB treatment. These plates correspond to the experimental results shown in Fig 4. Each image was taken 24 days after the initiation of treatment. The black circles at the center of each colony represent the original colony diameter immediately before the first treatment. Control colonies received no UVB or methoxsalen exposure, and demonstrate normal colony growth.
(TIF)

## References

[1] Holden WM, Fites JS, Reinert LK, Rollins-Smith LA. Nikkomycin Z is an effective inhibitor of the chytrid fungus linked to global amphibian declines. Fungal Biology. 2014 1 1;118(1):48–60. 24433676 10.1016/j.funbio.2013.11.001

[2] Valenzuela‐Sánchez A, O’Hanlon SJ, Alvarado‐Rybak M, Uribe‐Rivera DE, Cunningham AA, Fisher MC, et al Genomic epidemiology of the emerging pathogen Batrachochytrium dendrobatidis from native and invasive amphibian species in Chile. Transboundary & Emerging Diseases. 2018 4;65(2):309–14. 29205924 10.1111/tbed.12775

[3] Eskew EA, Todd BD. Parallels in amphibian and bat declines from pathogenic fungi. Emerg Infect Dis. 2013 3;19(3):379–85. 23622255 10.3201/eid1093.120707 PMC3647649

[4] Martel A, Vila‐Escale M, Fernández‐Giberteau D, Martinez‐Silvestre A, Canessa S, Praet SV, et al Integral chain management of wildlife diseases. Conservation Letters. 2020;13(2):e12707.

[5] Bernard RF, Evans J, Fuller NW, Reichard JD, Coleman JTH, Kocer CJ, et al Different management strategies are optimal for combating disease in East Texas cave versus culvert hibernating bat populations. Conservation Science and Practice. 2019;1(10):e106.

[6] Woodhams DC, Geiger CC, Reinert LK, Rollins-Smith LA, Lam B, Harris RN, et al Treatment of amphibians infected with chytrid fungus: learning from failed trials with itraconazole, antimicrobial peptides, bacteria, and heat therapy. Dis Aquat Org. 2012 2 17;98(1):11–25. 22422126 10.3354/dao02429

[7] Camp RJ, LaPointe DA, Hart PJ, Sedgwick DE, Canale LK. Large-scale tree mortality from Rapid Ohia Death negatively influences avifauna in lower Puna, Hawaii Island, USA. Condor. 2019 5 1;121(2).

[8] Loope L, Hughes F, Keith L, Harrington T, Hauff R, Friday JB, et al Guidance document for Rapid Ohia Death: background for the 2017–2019 ROD Strategic Response Plan. University of Hawaii: College of Tropical Agriculture and Human Resources 2016.

[9] White-nose syndrome [Internet]. [cited 2020 Apr 12]. Available from: https://www.usgs.gov/centers/nwhc/science/white-nose-syndrome?qt-science_center_objects=0#qt-science_center_objects

[10] Blehert DS, Hicks AC, Behr M, Meteyer CU, Berlowski-Zier BM, Buckles EL, et al Bat white-nose syndrome: an emerging fungal pathogen? Science. 2009 1 9;323(5911):227–227. 18974316 10.1126/science.1163874

[11] Frank CL, Davis AD, Herzog C. The evolution of a bat population with white-nose syndrome (WNS) reveals a shift from an epizootic to an enzootic phase. Frontiers in Zoology. 2019 12 3;16(1):40. 31827569 10.1186/s12983-019-0340-y PMC6889174

[12] Wibbelt G, Kurth A, Hellmann D, Weishaar M, Barlow A, Veith M, et al White-nose syndrome fungus (Geomyces destructans) in bats, Europe. Emerg Infect Dis. 2010 8;16(8):1237–43. 20678317 10.3201/eid1608.100002 PMC3298319

[13] Chaturvedi V, Springer DJ, Behr MJ, Ramani R, Li X, Peck MK, et al Morphological and molecular characterizations of psychrophilic fungus *Geomyces destructans* from New York bats with White Nose Syndrome (WNS). PLoS ONE. 2010 5 24;5(5):e10783. 20520731 10.1371/journal.pone.0010783 PMC2875398

[14] Thapa V, Turner GG, Hafenstein S, Overton BE, Vanderwolf KJ, Roossinck MJ. Using a novel partitivirus in *Pseudogymnoascus destructans* to understand the epidemiology of white-nose syndrome. PLOS Pathogens. 2016 12 27;12(12):e1006076. 28027325 10.1371/journal.ppat.1006076 PMC5189944

[15] Verant ML, Boyles JG, Jr WW, Wibbelt G, Blehert DS. Temperature-Dependent Growth of *Geomyces destructans*, the fungus that causes bat white-nose syndrome. PLOS ONE. 2012 9 28;7(9):e46280. 23029462 10.1371/journal.pone.0046280 PMC3460873

[16] Palmer JM, Drees KP, Foster JT, Lindner DL. Extreme sensitivity to ultraviolet light in the fungal pathogen causing white-nose syndrome of bats. Nature Communications. 2018 1 2;9(1):35. 10.1038/s41467-017-02441-z PMC5750222 29295979

[17] Marroquin CM, Lavine JO, Windstam ST. Effect of humidity on development of *Pseudogymnoascus destructans*, the causal agent of bat white-nose syndrome. nena. 2017 3;24(1):54–64.

[18] Verant ML, Meteyer CU, Speakman JR, Cryan PM, Lorch JM, Blehert DS. White-nose syndrome initiates a cascade of physiologic disturbances in the hibernating bat host. BMC Physiol. 2014 12 9;14:10. 25487871 10.1186/s12899-014-0010-4 PMC4278231

[19] Cryan PM, Meteyer CU, Boyles JG, Blehert DS. Wing pathology of white-nose syndrome in bats suggests life-threatening disruption of physiology. BMC Biology. 2010 11 11;8(1):135. 21070683 10.1186/1741-7007-8-135 PMC2984388

[20] Meteyer CU, Barber D, Mandl JN. Pathology in euthermic bats with white nose syndrome suggests a natural manifestation of immune reconstitution inflammatory syndrome. Virulence. 2012 11 15;3(7):583–8. 23154286 10.4161/viru.22330 PMC3545935

[21] Hoyt JR, Langwig KE, White JP, Kaarakka HM, Redell JA, Parise KL, et al Field trial of a probiotic bacteria to protect bats from white-nose syndrome. Scientific Reports. 2019 6 24;9(1):1–9. 31235813 10.1038/s41598-019-45453-z PMC6591354

[22] Rocke TE, Kingstad-Bakke B, Wüthrich M, Stading B, Abbott RC, Isidoro-Ayza M, et al Virally-vectored vaccine candidates against white-nose syndrome induce anti-fungal immune response in little brown bats (*Myotis lucifugus*). Sci Rep. 2019 5 1;9 (1) 10.1038/s41598-019-43210-w PMC6494898 31043669

[23] Santos E, Levitz SM. Fungal vaccines and immunotherapeutics. Cold Spring Harb Perspect Med. 2014 11;4(11). 10.1101/cshperspect.a019711 PMC4208708 25368016

[24] Hartman C, Goodrich S, Lange M, Coy Z, Cohen A, Mester J. Inactivation of fungal spores with heat and UVC, (Abstract). Presented at: Kentucky Academy of Science Annual Meeting; 2017; Murray, KY.

[25] Goodrich S, Hartman C, Lange M, Coy Z, Cohen A, Mester J. Inhibition of fungal colony growth by UVC, (Abstract). Presented at: Kentucky Academy of Science Annual Meeting; 2017; Murray, KY.

[26] Parrish JA, Fitzpatrick TB, Tanenbaum L, Pathak MA. Photochemotherapy of psoriasis with oral methoxsalen and longwave ultraviolet light. N Engl J Med. 1974 12;291(23):1207–11. 4422691 10.1056/NEJM197412052912301

[27] Filho OPS, Oliveira LAR, Martins FS, Borges LL, de Freitas O, Conceição EC da. Obtainment of pellets using the standardized liquid extract of *Brosimum gaudichaudii* Trécul (Moraceae). Pharmacognosy Magazine. 2015 1 1;11(41):170. 25709229 10.4103/0973-1296.149734 PMC4329620

[28] Hamerski D, Matern U. Elicitor-induced biosynthesis of psoralens in *Ammi majus* L. suspension cultures. European Journal of Biochemistry. 1988;171(1–2):369–75. 2828055 10.1111/j.1432-1033.1988.tb13800.x

[29] Gorgus E, Lohr C, Raquet N, Guth S, Schrenk D. Limettin and furocoumarins in beverages containing citrus juices or extracts. Food Chem Toxicol. 2010 1;48(1):93–8. 19770019 10.1016/j.fct.2009.09.021

[30] Grundmann-Kollmann M, Podda M, Bräutigam L, Hardt-Weinelt K, Ludwig RJ, Geisslinger G, et al Spatial distribution of 8-methoxypsoralen penetration into human skin after systemic or topical administration. British Journal of Clinical Pharmacology. 2002 11;54(5):535–9. 12445034 10.1046/j.1365-2125.2002.01692.x PMC1874477

[31] Wolf P. Psoralen-ultraviolet A endures as one of the most powerful treatments in dermatology: reinforcement of this “triple-product therapy” by the 2016 British guidelines. Br J Dermatol. 2016 1;174(1):11–4. 26790646 10.1111/bjd.14341

[32] Stern RS, Nichols KT, Väkevä LH. Malignant melanoma in patients treated for psoriasis with methoxsalen (psoralen) and ultraviolet A radiation (PUVA). The PUVA Follow-Up Study. N Engl J Med. 1997 4 10;336(15):1041–5. 9091799 10.1056/NEJM199704103361501

[33] Amitay-Laish I, Prag-Naveh H, Dalal A, Pavlovsky L, Feinmesser M, Hodak E. Treatment of early folliculotropic mycosis fungoides with special focus on psoralen plus ultraviolet A. Acta Dermato-Venereologica. 2018 Nov;98(10):951–5. 10.2340/00015555-3013 30085321

[34] Archier E, Devaux S, Castela E, Gallini A, Aubin F, Le Maître M, et al Efficacy of psoralen UV-A therapy vs. narrowband UV-B therapy in chronic plaque psoriasis: a systematic literature review. J Eur Acad Dermatol Venereol. 2012 5;26 Suppl 3:11–21. 22512676 10.1111/j.1468-3083.2012.04519.x

[35] de Gruijl FR. Photocarcinogenesis: UVA vs UVB. Meth Enzymol. 2000;319:359–66. 10907526 10.1016/s0076-6879(00)19035-4

[36] de Menezes HD, Pereira AC, Brancini GTP, de Leão HC, Massola Júnior NS, Bachmann L, et al Furocoumarins and coumarins photoinactivate *Colletotrichum acutatum* and *Aspergillus nidulans* fungi under solar radiation. Journal of Photochemistry and Photobiology B: Biology. 2014 2 5;131:74–83. 10.1016/j.jphotobiol.2014.01.008 24509069

[37] Grossweiner LI. Mechanisms of photosensitization by furocoumarins. Natl Cancer Inst Monogr. 1984 12;66:47–54. 6397692

[38] Serrano-Pérez JJ, Merchán M, Serrano-Andrés L. Photoreactivity of furocoumarins and DNA in PUVA therapy: formation of psoralen−thymine adducts. J Phys Chem B. 2008 11 6;112(44):14002–10. 18855465 10.1021/jp805523d

[39] Timonen JM, Vuolteenaho K, Leppänen T, Nieminen RM, Aulaskari P, Jänis J, et al Synthesis of novel anti-inflammatory psoralen derivatives—structures with distinct anti-inflammatory activities. Journal of Heterocyclic Chemistry. 2018;55(11):2590–7.

[40] Hermanson G. Bioconjugate Techniques. 3rd ed. Elsevier; 2013.

[41] Kitamura N, Kohtani S, Nakagaki R. Molecular aspects of furocoumarin reactions: Photophysics, photochemistry, photobiology, and structural analysis. Journal of Photochemistry and Photobiology C: Photochemistry Reviews. 2005 10 1;6(2):168–85.

[42] Parsons BJ. Psoralen photochemistry. Photochemistry and Photobiology. 1980;32(6):813–21. 7005917 10.1111/j.1751-1097.1980.tb04061.x

[43] Lai C, Cao H, Hearst JE, Corash L, Luo H, Wang Y. Quantitative analysis of DNA interstrand cross-links and monoadducts formed in human cells induced by psoralens and UVA irradiation. Anal Chem. 2008 11 15;80(22):8790–8. 18947205 10.1021/ac801520m PMC2626160

[44] Hearst JE, Isaacs ST, Kanne D, Rapoport H, Straub K. The reaction of the psoralens with deoxyribonucleic acid. Quarterly Reviews of Biophysics. 1984 2;17(1):1–44. 6385057 10.1017/s0033583500005242

[45] Schmidt BR, Geiser C, Peyer N, Keller N, Von Rütte M. Assessing whether disinfectants against the fungus *Batrachochytrium dendrobatidis* have negative effects on tadpoles and zooplankton. Amphibia-Reptilia. 2009 Aug;30(3):313–9.

[46] PubChem. Sodium hypochlorite [Internet]. [cited 2020 Jun 22]. Available from: https://pubchem.ncbi.nlm.nih.gov/compound/23665760

[47] PubChem. Psoralen [Internet]. [cited 2020 Jun 22]. Available from: https://pubchem.ncbi.nlm.nih.gov/compound/6199

[48] PubChem. 8-Methoxypsoralen [Internet]. [cited 2020 Jul 8]. Available from: https://pubchem.ncbi.nlm.nih.gov/source/hsdb/2505

[49] Dunnick JK. Toxicology and carcinogenesis studies of 8-methoxypsoralen in F344/N rats 1989 Research Triangle Park, NC: National Toxicology Program. NTP TR 359. 12695782

[50] Lowe NJ, Weingarten D, Bourget T, Moy LS. PUVA therapy for psoriasis: Comparison of oral and bath-water delivery of 8-methoxypsoralen. Journal of the American Academy of Dermatology. 1986 5 1;14(5, Part 1):754–60. 3711379 10.1016/s0190-9622(86)70089-3

[51] Gasparro FP. The role of PUVA in the treatment of psoriasis. Photobiology issues related to skin cancer incidence. Am J Clin Dermatol. 2000;1(6):337–48. 11702610 10.2165/00128071-200001060-00002

[52] Silva EBF, Barbosa IJF, Barreto HM, Siqueira-Júnior JP. Modulation of the UVB-induced lethality by furocoumarins in *Staphylococcus aureus*. Journal of Photochemistry and Photobiology B: Biology. 2014 1 5;130:260–3. 10.1016/j.jphotobiol.2013.11.012 24362322

[53] Brasch J, Kay C. Effects of repeated low-dose UVB irradiation on the hyphal growth of Candida albicans. Mycoses. 2006 1;49(1):1–5. 10.1111/j.1439-0507.2005.01169.x 16367810

[54] Begum M, Hocking AD, Miskelly D. Inactivation of food spoilage fungi by ultra violet (UVC) irradiation. Int J Food Microbiol. 2009 1;129(1):74–7. 19059664 10.1016/j.ijfoodmicro.2008.11.020

[55] WHO | UV radiation [Internet]. WHO. World Health Organization; [cited 2020 Mar 13]. Available from: http://www.who.int/uv/faq/whatisuv/en/

[56] Boyce JM. Modern technologies for improving cleaning and disinfection of environmental surfaces in hospitals. Antimicrobial Resistance & Infection Control. 2016 4 11;5(1):10. 27069623 10.1186/s13756-016-0111-x PMC4827199

[57] Mustapha A, Alhmidi H, Cadnum JL, Jencson AL, Donskey CJ. Efficacy of manual cleaning and an ultraviolet C room decontamination device in reducing health care-associated pathogens on hospital floors. Am J Infect Control. 2018;46(5):584–6. 29306489 10.1016/j.ajic.2017.10.025

[58] Kim D-K, Kang D-H. Elevated Inactivation Efficacy of a Pulsed UVC Light-Emitting Diode System for Foodborne Pathogens on Selective Media and Food Surfaces. Elkins CA, editor. Appl Environ Microbiol. 2018 10 15;84(20):e01340–18. 30097449 10.1128/AEM.01340-18 PMC6182902

[59] Pfeifer GP, You Y-H, Besaratinia A. Mutations induced by ultraviolet light. Mutation Research/Fundamental and Molecular Mechanisms of Mutagenesis. 2005 4 1;571(1–2):19–31. 15748635 10.1016/j.mrfmmm.2004.06.057

[60] Borderie F, Alaoui-Sehmer L, Bousta F, Alaoui-Sossé B, Aleya L. Cellular and molecular damage caused by high UV-C irradiation of the cave-harvested green alga Chlorella minutissima: Implications for cave management. International Biodeterioration & Biodegradation. 2014 9 1;93:118–30.

[61] Collecting, preserving and shipping specimens for the diagnosis of avian influenza A(H5N1) virus infection, Guide for field operations, World Health Organization. 2006 10;39–41.

[62] Bond CA, Grant K, Boh L. Photochemotherapy of psoriasis with methoxsalen and longwave ultraviolet light (PUVA). Am J Hosp Pharm. 1981 Jul;38(7):990–5. 7020414

[63] Oxsoralen-Ultra (Methoxsalen Capsules): Uses, Dosage, Side Effects, Interactions, Warning [Internet]. RxList. [cited 2020 Jun 22]. Available from: https://www.rxlist.com/oxsoralen-ultra-drug.htm

[64] Stern RS, Lange R. Outcomes of pregnancies among women and partners of men with a history of exposure to methoxsalen photochemotherapy (PUVA) for the treatment of psoriasis. Arch Dermatol. 1991 3;127(3):347–50. 1998364

[65] Oginsky EL, Green GS, Griffith DG, Fowlks WL. Lethal photosensitization of bacteria with 8-methoxypsoralen to long wave length ultraviolet radiation. J Bacteriol. 1959 12;78(6):821–33. 14428425 10.1128/jb.78.6.821-833.1959 PMC290639

[66] Xiao Y, Chu XN, He M, Liu XC, Hu JY. Impact of UVA pre-radiation on UVC disinfection performance: Inactivation, repair and mechanism study. Water Res. 2018 9 15;141:279–88. 29800836 10.1016/j.watres.2018.05.021

[67] Gibbs NK, Quanten E, Baydoun S, Knox CN, Roelandts R, De Schryver F, et al Photophysical, photochemical and photobiological properties of pyrrolocoumarins; A new class of photoactive compounds. Journal of Photochemistry and Photobiology B: Biology. 1988 7 1;2(1):109–22. 10.1016/1011-1344(88)85040-1 3149297

[68] Turner GG, Meteyer CU, Barton H, Gumbs JF, Reeder DM, Overton B, et al Nonlethal screening of bat-wing skin with the use of ultraviolet fluorescence to detect lesions indicative of white-nose syndrome. Journal of Wildlife Diseases. 2014 5 22;50(3):566–73. 24854396 10.7589/2014-03-058

[69] Eichenfield LF, Tom WL, Chamlin SL, Feldman SR, Hanifin JM, Simpson EL, et al Guidelines of care for the management of atopic dermatitis: section 1. Diagnosis and assessment of atopic dermatitis. J Am Acad Dermatol. 2014 2;70(2):338–51. 24290431 10.1016/j.jaad.2013.10.010 PMC4410183

[70] Wentworth A, Hartman C, Cohen A, Mester J. Treatment of fungi with photosensitizers and UVA (Abstract). Presented at: Association of Southeastern Biologists Annual Meeting; 2019; Memphis, TN.

