# Supplementary figures and images for: Novel inactivation of the causative fungal pathogen of white-nose syndrome with methoxsalen plus ultraviolet A or B radiation
Source: PLoS One. 2020 Sep 11;15(9):e0239001. doi: 10.1371/journal.pone.0239001 (PMC7485863; doi:10.1371/journal.pone.0239001)

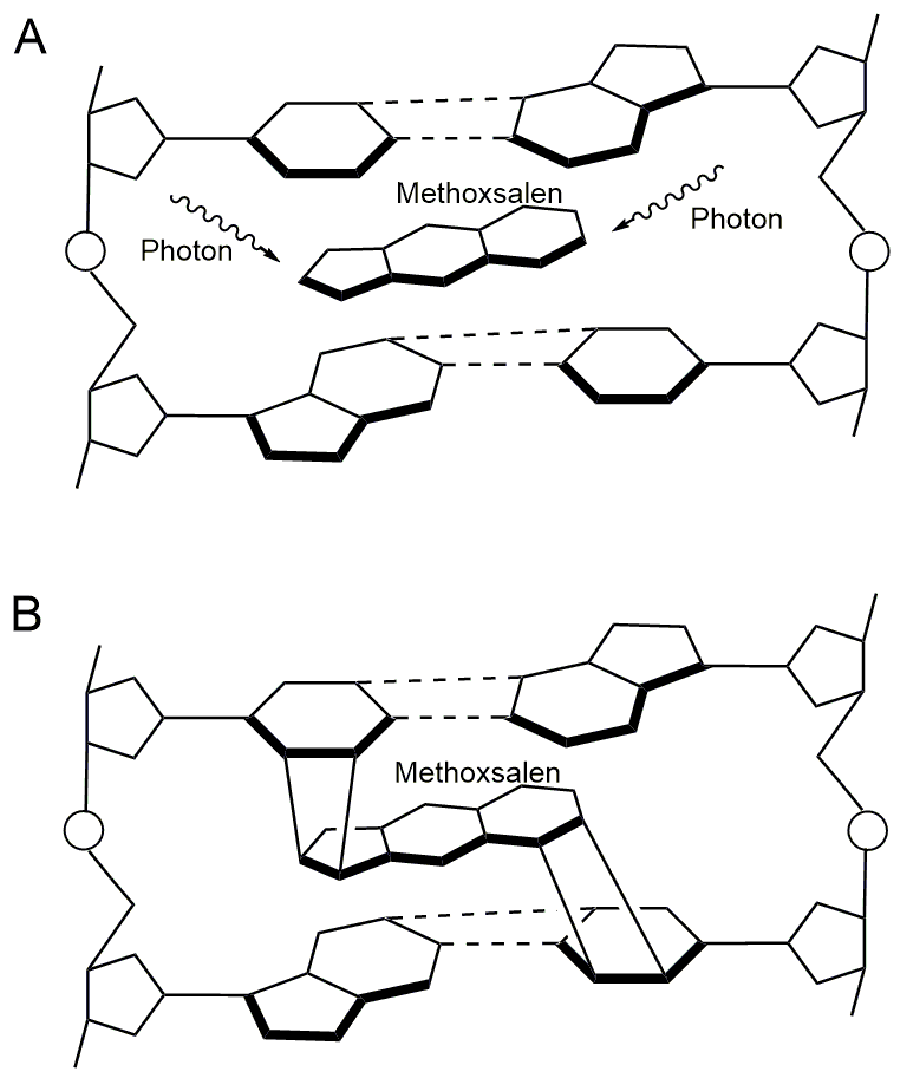

Supplement: S1 Fig — (A) Intercalation of methoxsalen between DNA base pairs. (B) Covalent crosslinks form when activated by UV radiation. (TIF) [file pone.0239001.s001.tif]

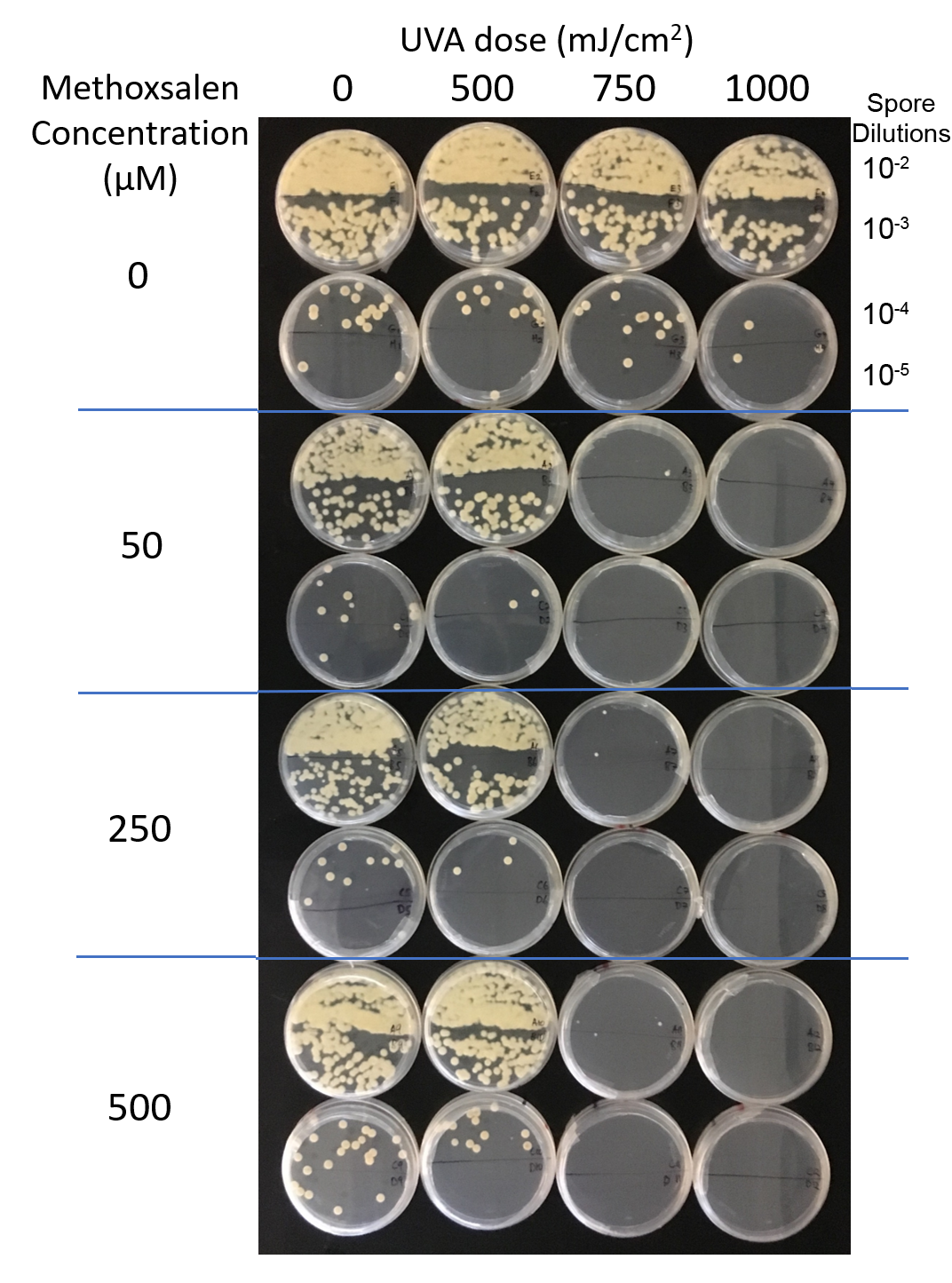

Supplement: S2 Fig — Spore germination, resulting in P. destructans colony formation, was observed after various treatments. Each set of 2 rows represents spore dilutions of 10−2 to 10−5 split across two plates. Each column shows spores exposed to a specific UVA dose. Each set of 2 rows represents spores pre-treated with a specific amount of methoxsalen (the first 2 rows show spore controls that were not pre-treated with methoxsalen). Spores were suspended in various concentrations of methoxsalen for 20–24 hours before UVA exposure. Spore inactivation was evident for all spores that were treated with both methoxsalen and UVA. This image was taken 11 days after spore plating. (TIF) [file pone.0239001.s002.tif]

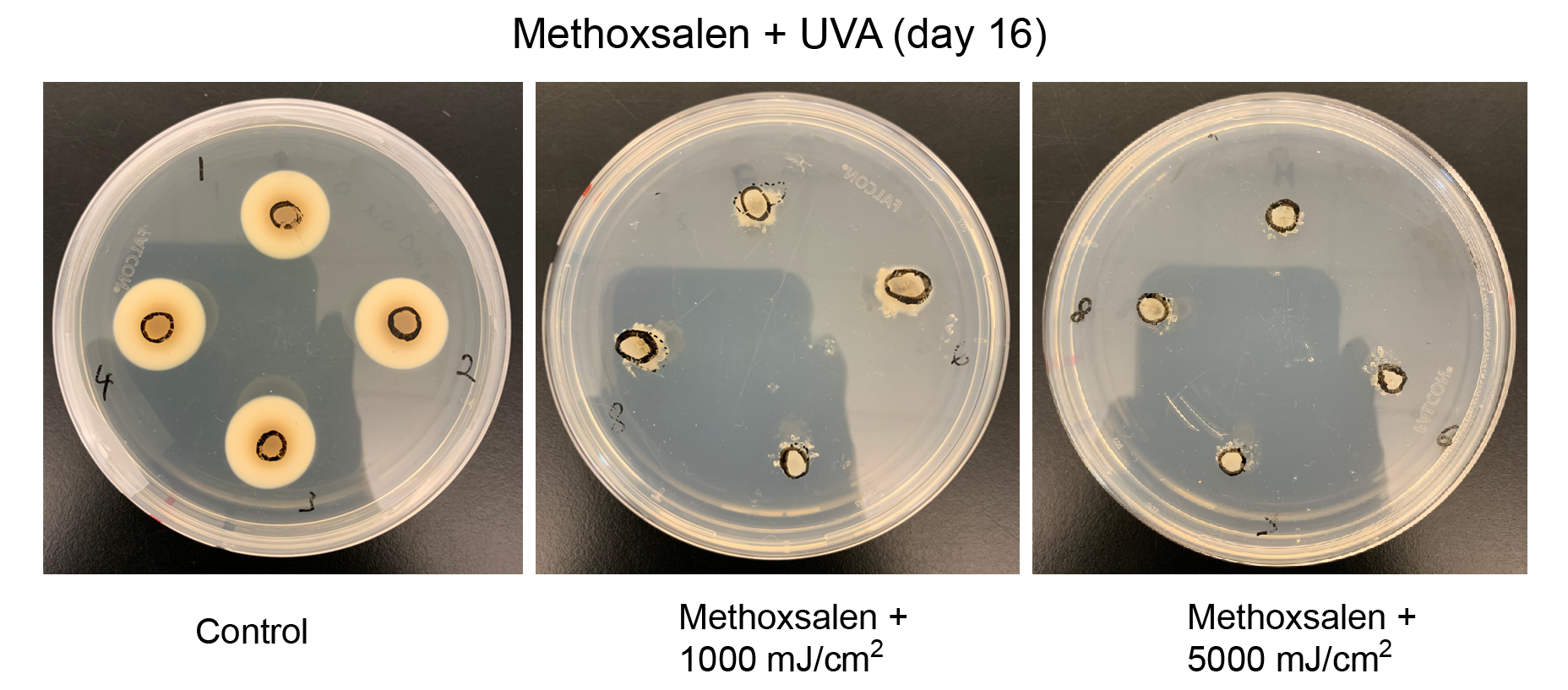

Supplement: S3 Fig — These plates correspond to the experimental results shown in Fig 3. Each image was taken 16 days after the initiation of treatment. The black circles at the center of each colony represent the original colony diameter immediately before the first treatment. The control plate shows normal colony growth. (TIF) [file pone.0239001.s003.tif]

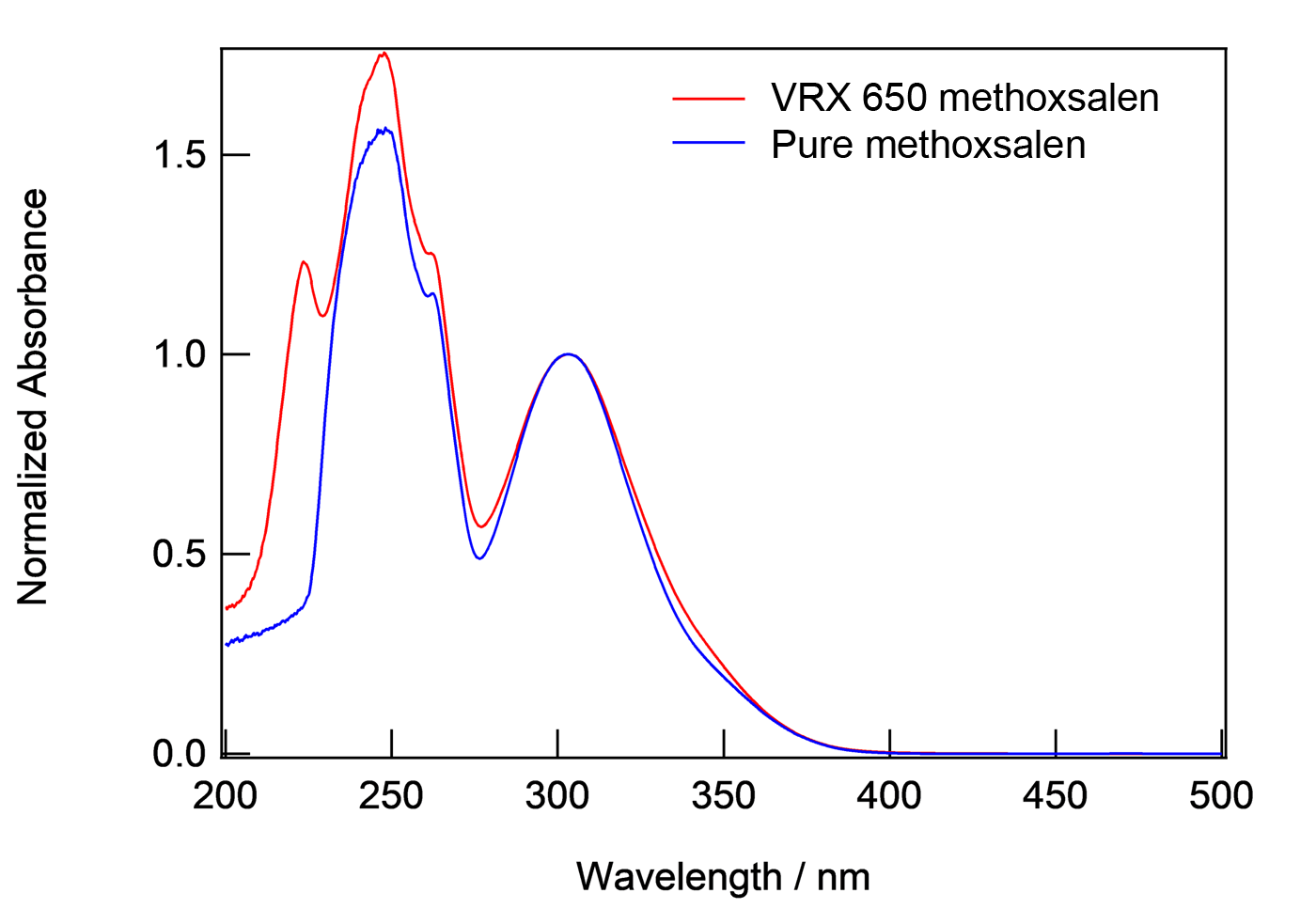

Supplement: S4 Fig — Solvent-subtracted normalized absorption spectra of methoxsalen extracted from VRX 650 capsules (red) and purified methoxsalen powder (blue). (TIF) [file pone.0239001.s004.tif]

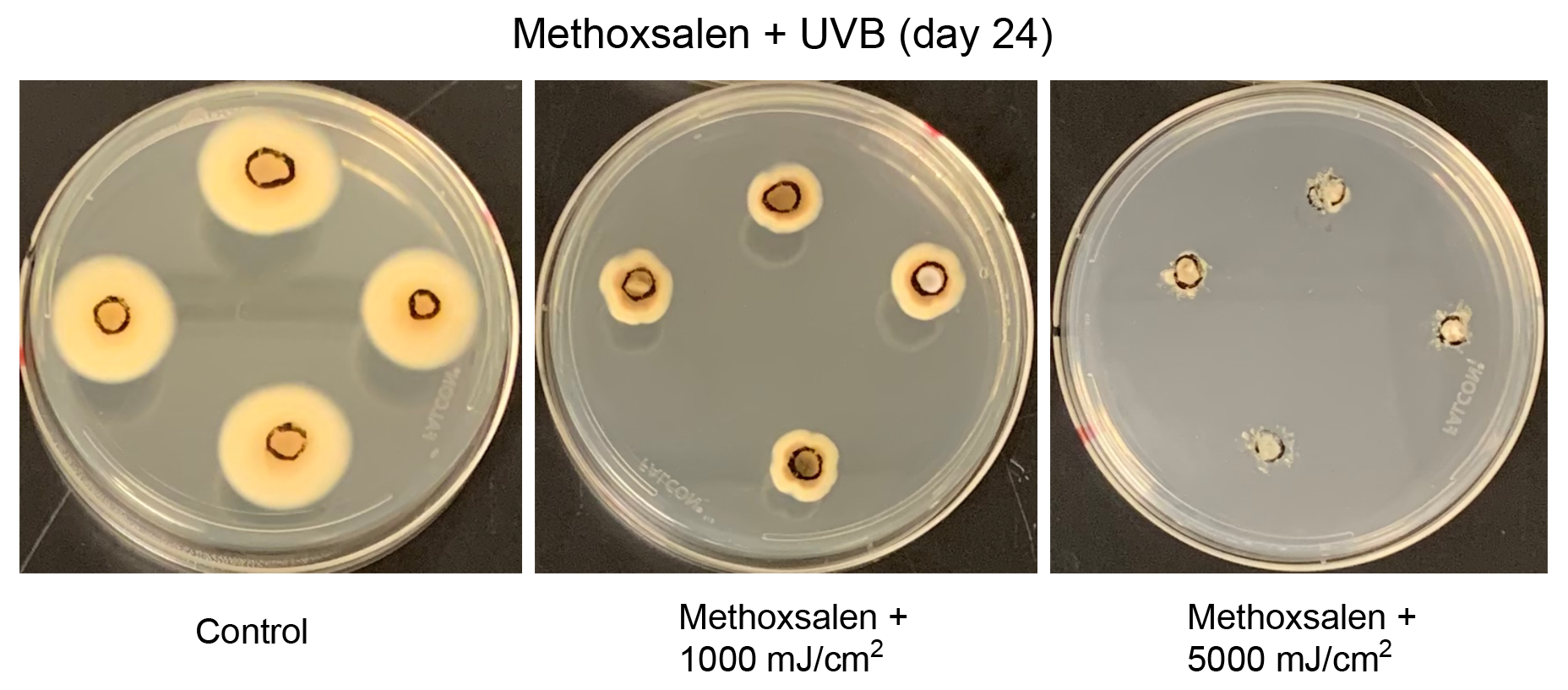

Supplement: S5 Fig — These plates correspond to the experimental results shown in Fig 4. Each image was taken 24 days after the initiation of treatment. The black circles at the center of each colony represent the original colony diameter immediately before the first treatment. Control colonies received no UVB or methoxsalen exposure, and demonstrate normal colony growth. (TIF) [file pone.0239001.s005.tif]
